# Mechanisms of Increased Indoxacarb Toxicity in Methoxyfenozide-Resistant Cotton Bollworm *Helicoverpa armigera* (Lepidoptera: Noctuidae)

**DOI:** 10.3390/toxics8030071

**Published:** 2020-09-17

**Authors:** Qinqin Wang, Changhui Rui, Qiyuan Wang, Li Wang, Fugen Li, Shahzad Ali Nahiyoon, Huizhu Yuan, Li Cui

**Affiliations:** 1 Key Laboratory of Integrated Pest Management in Crops, Institute of Plant Protection, Chinese Academy of Agricultural Sciences, Ministry of Agriculture, Beijing 100193, China; 82101189121@caas.cn (Q.W.); chrui@ippcaas.cn (C.R.); wangqiyuan2589@outlook.com (Q.W.); 82101172327@caas.cn (L.W.); Shezi_129@yahoo.com (S.A.N.); hzhyuan@ippcaas.cn (H.Y.); 2 Institute for the Control of Agrochemicals, Ministry of Agriculture and Rural Affairs, Beijing 100125, China; lifugen@agri.gov.cn

**Keywords:** indoxacarb, activation toxicity, methoxyfenozide, negative cross-resistance, detoxification enzymes, *Helicoverpa armigera*

## Abstract

Indoxacarb is an important insecticide for the selective control of *Helicoverpa armigera*. It can be bioactivated to the more effective N-decarbomethoxylated indoxacarb (DCJW) by esterases in pests. It was observed that both field and laboratory selected populations of *H. armigera* showed negative cross-resistance between indoxacarb and methoxyfenozide. The Handan population exhibited moderate resistance to indoxacarb, but was susceptible to methoxyfenozide; the Baoding and Yishui populations exhibited moderate resistance to methoxyfenozide, but they were susceptible to indoxacarb. Moreover, the toxicity of indoxacarb was enhanced 1.83-fold in the laboratory methoxyfenozide-resistant *H. armigera*, and susceptibility to methoxyfenozide was increased 2.81-fold in the laboratory indoxacarb-resistant *H. armigera*. In vivo, DCJW concentrations in the susceptible and methoxyfenozide-selected (laboratory methoxyfenozide-resistant) populations were 4.59- and 4.31-fold greater than in the indoxacarb-resistant Handan population 1 h after dosing. After 2 h, the highest concentrations of DCJW and indoxacarb appeared in the methoxyfenozide-selected population. Meanwhile, increased carboxyl esterase (CarE) and decreased glutathione S-transferase (GST) activities were observed in the methoxyfenozide-selected population. However, the indoxacarb-selected (laboratory indoxacarb-resistant) and Handan populations showed a higher disappearance of indoxacarb and DCJW, and the activity of cytochrome P450 mono-oxygenase in these populations were significantly increased. This study showed that the improved toxicity of indoxacarb, as observed in the methoxyfenozide-selected *H. armigera*, was correlated with increased CarE activity, decreased GST activity, and the in vivo accumulation of indoxacarb and DCJW. The significantly increased cytochrome P450 activity and higher disappearance of indoxacarb and DCJW in indoxacarb-resistant *H. armigera* resulted in the decreased toxicity of indoxacarb.

## 1. Introduction

Cotton bollworm *Helicoverpa armigera* (Hübner; Lepidoptera: Noctuidae) is a global agricultural pest that damages a huge variety of cultivated crops, such as maize, cotton, tomatoes, and wheat [1]. The larvae of *H. armigera* prefer to make holes and feed on blooms, flower heads, and fruits. Its pest status is enhanced by its high fecundity, strong ability to migrate long distances, highly polyphagous nature, and enhanced ability to develop resistance to a variety of insecticide classes [2,3]. This pest’s increased resistance to conventional insecticides such as organophosphates, organochlorines, pyrethroids, and carbamates, makes the implementation of a comprehensive and tailor-made resistance-management program of primary importance [4].

To adequately control this pest, various classes of insecticides with different modes of action have been used, including indoxacarb and methoxyfenozide. Indoxacarb is a pyrazoline-type insecticide with low mammalian toxicity and high insecticidal activity [5,6,7]. This insecticide shows excellent field activity against the lepidopteran and coleopteran pests of cotton, corn, peanuts, soybeans, vegetables, fruits, and other crops [8]. Metabolic studies showed that indoxacarb can be bioactivated to the active N-decarbomethoxylated indoxacarb (DCJW) metabolite by esterases [5,6,9]. Indoxacarb and its activated metabolite DCJW have a novel mode of action that involves the blocking of sodium channels, which results in the paralysis and death of the targeted species [6]. Moreover, activated metabolite DCJW is more effective in blocking insect sodium channels than its parent compound, indoxacarb. Although indoxacarb is prone to be bioactivated to DCJW in lepidopteran insects [10], resistance to indoxacarb was documented in a broad range of insect pests, such as *Musca domestica*, *Spodoptera litura,* and *Plutella xylostella* [11,12,13,14,15]. *H. armigera* has also developed resistance to indoxacarb, which was reported in China [16].

Cross-resistance among insecticides is a common concern in insect-pest management. To delay the development of resistance, it is important to determine the underlying resistance mechanisms. Metabolic mechanisms, such as increased activities of esterases, glutathione S-transferases (GSTs), and cytochrome P450s, can contribute to cross-resistance development [17,18,19]. Some reports revealed that cross-resistance exists between indoxacarb and metaflumizone in *P. xylostella* because they are both sodium-channel-blocker insecticides that have the same target site [20,21]. Similarly, cross-resistance between cypermethrin and indoxacarb in *P. xylostella* was studied. Nehare et al. reported that resistance to cypermethrin in an indoxacarb-resistant strain could be conferred by the elevated activity of esterases in resistant strains or the location of the resistant gene on the same chromosome [15,19]. Timely pesticide-application strategies can delay the development of cross-resistance, such as the rotation of insecticides with novel mechanisms [18]. Methoxyfenozide is an ecdysone agonist that can be used as a biorational insecticide for controlling various insect pests, including *H. armigera*. It was reported that LC_50_ values of indoxacarb in *S. litura* were negatively correlated with LC_50_ values of abamectin and methoxyfenozide [22]. However, the negative correlation between indoxacarb and methoxyfenozide in *H. armigera* was unclear. Therefore, the mechanisms of negative correlation between indoxacarb and methoxyfenozide should also be investigated.

In this work, the susceptibility of different populations of *H. armigera* to indoxacarb and methoxyfenozide was determined. We also evaluated the metabolic activation of indoxacarb to DCJW in four populations of *H. armigera* with varying susceptibility levels to indoxacarb and methoxyfenozide. Lastly, detoxification-enzyme activities in the larval midgut were also measured.

## 2. Materials and Methods

### 2.1. H. armigera Populations

A susceptible (SUS) population of *H. armigera*, originally collected from the main cotton growing areas of China, was reared in the laboratory without insecticide exposure. An indoxacarb-selected (IND-SEL) population was developed from the SUS population via selection with indoxacarb for 13 generations. A methoxyfenozide-selected (MET-SEL) population was developed from the SUS population via selection with methoxyfenozide for 30 generations. Individuals of field populations were collected from cotton fields in the cities of Handan (36.86 N, 115.22 E), Langfang (39.51 N, 116.61 E), Baoding (38.85 N, 115.48 E), Yishui (35.78 N, 118.64 E), and Anqiu (36.49 N, 119.28 E) (the main cotton growing areas), China in June 2017. Larvae of all populations were reared on an artificial diet. All larvae were maintained on an artificial diet (each 1500 g of artificial diet contained 100 g soy flour, 300 g corn flour, 100 g beer yeast, 1.5 g vitamin, 15 g agar, 10 g ascorbic acid, 1 g sorbic acid, 2.5 citric acid, 0.0625 g erythrocin, and propionic acid solution (100 mL distilled water and 5 mL propionic acid)). *H. armigera* populations were maintained in an environment of 25 ± 2 °C and 60–70% relative humidity with a 14:10 light/dark photoperiod. Adults were maintained under the same conditions of temperature and light and were fed a 10% honey solution.

### 2.2. Chemicals

Indoxacarb (95%) was provided by DuPont Company (Wilmington, DE, USA). Methoxyfenozide (98.2%) was obtained from Dow Agro Sciences (Zionsville, IN, USA). Indoxacarb (99.6%) and DCJW (99%) were kindly provided by Fugen Li. Ultraperformance liquid chromatography (UPLC)-grade acetonitrile was purchased from Merck Co., Rahway, NJ, USA. Analytical-grade ammonium acetate, ethanol, ethyl acetate, acetone, and dimethylformamide (DMF) were obtained from Beijing Chemical Reagents Co., Beijing, China. Bovine serum albumin (BSA), Coomassie brilliant blue G-250, α-naphthyl acetate (α-NA), sodium dodecyl sulfate (SDS), 2,4-dinitrochlorobenzene (CDNB), glutathione (GSH), and dithionitrobenzoic acid (DTNB) were purchased from Sigma Chemical Corporation (St. Louis, MO, USA). Insect multifunction oxidase ELISA kit was purchased from Huabaitai Biotechnology Corporation (Beijing, China).

### 2.3. Indoxacarb and Methoxyfenozide Toxicities to H. armigera

The toxicities of indoxacarb and methoxyfenozide to *H. armigera* were determined using the leaf-dipping method. Stock indoxacarb and methoxyfenozide solutions were prepared and diluted using a 0.05% (w/v) Triton X-100 aqueous solution to 5–7 concentrations. Bioassays were performed with three replicates for each concentration (20 larvae for each replicate). Pak choi (*Brassica rapa chinensis*) leaf discs (2 cm diameter) were treated with the solution for 10 s and dried on tissue paper. Then, individual leaf discs were transferred to 10-well culture plates. Control larvae were treated with an aqueous solution of 0.05% Triton X-100, and mortality was less than 10%. The larvae were fed the treated leaf discs, and reared under the conditions described above. The mortality of the larvae was recorded at 48 and 72 h after indoxacarb and methoxyfenozide treatment, respectively [23]. Larvae that did not move when touched with a brush were considered dead.

### 2.4. In Vitro Indoxacarb Metabolism

#### 2.4.1. Crude-Enzyme Preparation from *H. armigera Midgut*

Sixth-instar larvae were dissected on ice, and the midgut of each larva was gently shaken, rinsed in cold 1.15% KCl aqueous solution, and dried on absorbent paper. Then, the midgut was homogenized on ice in 2 mL of 0.1 mol L^−1^ ammonium acetate (CH_3_COONH_4_) solution. After homogenization, the homogenate was centrifuged at 4 °C and 10,000× *g* for 10 min, and the supernatant was used as an enzyme source for the determination of protein concentration, metabolic, and detoxification-enzyme activities [9]. Each enzyme sample was derived from ten individuals of *H. armigera.*

#### 2.4.2. Indoxacarb N-Decarbomethoxylation Reaction

The *H. armigera* midgut was shown to be highly capable of activating indoxacarb [6]. Three milliliters of the incubation mixture was used to obtain a high concentration of DCJW. The mixture consisted of 40 μL of 90 mmoL/L indoxacarb solutions, and 1.96 mL of 0.1 mol L^−1^ ammonium acetate (CH_3_COONH_4_) aqueous solution (pH 6.8). Then, one milliliter of the enzyme source was added to start the reaction. After incubation at 30 °C with a constant temperature oscillator for 1 or 2 h, 3 mL of ice-cold ethyl acetate (CH_3_COOC_2_H_5_) was added to stop the reactions. The reactions of the control samples were stopped by adding 3 mL of cold CH_3_COOC_2_H_5_ at 0 h. Then, the water phase was extracted with 2 and 1 mL CH_3_COOC_2_H_5_. The CH_3_COOC_2_H_5_ phase was combined and dried under pure N_2_ at 20 °C, and then resuspended in 500 μL of acetonitrile. The sample was filtered through a 0.22 μm membrane for ultraperformance liquid chromatography (UPLC) analysis [9]. The enzyme control (no substrate) and substrate control (no enzyme sample) were also prepared. Metabolite activity was expressed as pmol DCJW min^−1^ mg^−1^ protein.

#### 2.4.3. Ultraperformance Liquid Chromatography Analysis

Chromatographic separation was conducted using an ACQUITY UPLC BEH C-18 column (2.1 by 50 mm, 1.7 μm) with a column temperature of 30 °C. Samples were eluted at a constant flow rate of 0.2 mL min^−1^ using a mobile phase consisting of acetonitrile and water (80:20). The eluate was monitored by an ultraviolet detector set at 314 nm [10,13,24]; injection volume was 2 μL. The peaks of indoxacarb and DCJW were integrated. The peak area of DCJW was used for least-squares linear-regression analysis. Standard solutions of pure DCJW ranging from 0.1 to 1000 μg L^−1^ were analyzed to evaluate the linearity of the regression. The slope and correlation coefficient (R^2^) of the linear-regression equation were calculated.

#### 2.4.4. DCJW Recovery

Method sensitivity was determined on the basis of the limit of quantitation (LOQ), which was estimated as the minimal spiked concentration with suitable recovery (70% < R < 120%) and precision (relative standard deviation (RSD) < 20%) [25]. To evaluate the accuracy and precision of this developed UPLC method of DCJW, recovery studies were carried out. For the recovery assays, enzyme control (no substrate sample) was spiked with standard solutions of DCJW at 40, 200, and 1000 μg L^−1^. Afterward, the extraction was processed according to the aforementioned method. Each sample was filtered through a 0.22 μm membrane for UPLC analysis. The accuracy of this analytic method was determined from the recovery ratio (RE) and the RSD: RE% = concentration found × 100/known concentration.

### 2.5. Enzymes and Protein-Concentration Assays

The enzyme samples from the midgut of different populations of *H. armigera* were diluted using 66 mM phosphate buffer at pH 7.0 (GST assay) and 0.04 mol L^−1^ phosphate buffer at pH 7.0 (CarE assay). The protein concentration of each enzyme sample was analyzed following the method of Bradford, using bovine serum albumin (BSA) as the standard [26].

#### 2.5.1. Carboxyl Esterase (CarE) Assay

CarE activity was measured using the Van Asperen method with modifications [26]. The total substrate solution (5 mL) contained 10^−4^ mol L^−1^ physostigmine and 0.03 mol L^−1^ α-NA. Then, the enzyme sample (0–0.5 mL, diluted 20-fold) and phosphate-buffered saline (1–0.5 mL, 0.04 mol/L, pH 7.0) were added to the substrate solution, and the reaction mixtures were incubated in a shaking incubator for 30 min at 30 °C. One milliliter of a mixture of distilled water, SDS (35.7 mg), and fast blue B salt (2.9 mg) were added to stop the reaction. After 30 min, absorbance at 600 nm was examined using a Synergy HT multimode microplate reader. The results are shown as ΔOD_600_ min^−1^ mg^−1^ protein. Each enzyme sample was obtained from ten individuals, and three biological replicates were performed.

#### 2.5.2. GST Assay

GST activity was tested using the 2,4-dinitrochlorobenzene (CDNB) method. Enzyme samples (0.2 mL) were incubated with phosphate-buffered saline (66 mmol L^−1^, pH 7.0, PBS 2.4 mL), CDNB (30 mmol L^−1^, 0.1 mL), and GSH (50 mmol L^−1^, 0.3 mL). The Synergy HT multimode microplate reader at 27 °C and 340 nm with the kinetic mode was used to measure enzyme activities [27,28]. The results are shown as ΔOD_340_ min^−1^ mg^−1^ protein.

#### 2.5.3. Cytochrome P450 Assay

An insect multifunction oxidase ELISA kit was used to determine cytochrome P450 enzyme activity. According to the manufacturer’s instructions, fivefold diluted enzyme samples were added to the ELISA Microlon plates. Lastly, optical density (OD) values of the samples were tested at 450 nm [29]. The results are shown as ΔOD_450_ min^−1^ mg^−1^ protein.

### 2.6. Statistical Analysis

LC_50_ values and their 95% confidence limits (CLs) were calculated through probit analysis using SPSS 20.0 software (SPSS Inc., Chicago, IL, USA). Graphs were constructed using Origin 8.0 software. Resistance levels were classified according to the resistance ratio (RR) described by Lai et al. [30]: RR < 5, susceptible; RR = 5–10, low resistance level; RR = 10–40, moderate resistance level; RR = 40–160, high resistance level; and RR > 160, very high resistance level. N-decarbomethoxylation activity = amount of DCJW (pmol)/protein (mg) x time (min); the lowest N-decarbomethoxylation activity was observed in the HD population 1 h after dosing. Therefore, the HD population was used as the baseline. Activity ratio = N-decarbomethoxylation activity of other populations/N-decarbomethoxylation activity of the HD population. One-way analysis of variance (ANOVA), followed by Fisher’s least-significant-difference (LSD) test (α = 0.05), was used to compare indoxacarb N-decarbomethoxylation activities and enzyme activities in different *H. armigera* populations.

## 3. Results

### 3.1. Susceptibility of H. armigera Field Populations to Indoxacarb and Methoxyfenozide

**Figure 1:**
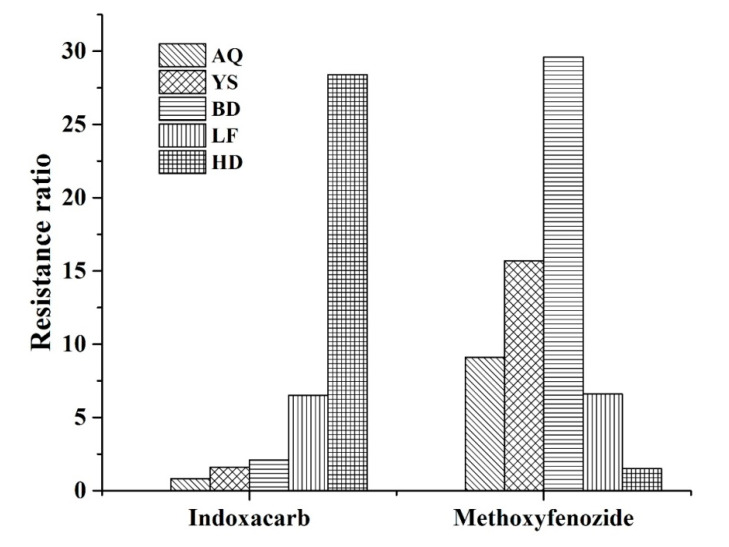
Resistance ratios of *H. armigera* field populations to indoxacarb and methoxyfenozide. AQ, Anqiu population; YS, Yishui population; BD, Baoding population; LF, Langfang population; HD, Handan population.

The susceptibility of field *H. armigera* to indoxacarb and methoxyfenozide is presented in Figure 1. The Handan population showed moderate resistance to indoxacarb (LC_50_ value, 176.9 mg L^−1^; resistance ratio, 28.4), but it was susceptible to methoxyfenozide (LC_50_ value, 128.2 mg L^−1^; resistance ratio, 1.5). The Baoding and Yishui populations showed medium resistance to methoxyfenozide (resistance ratios, 29.6 and 15.7, respectively), but they were susceptible to indoxacarb (resistance ratios, 2.1 and 1.6, respectively). However, the Langfang population showed a low level of resistance to both indoxacarb (resistance ratio, 6.5) and methoxyfenozide (resistance ratio, 6.6).

### 3.2. Toxicities of Indoxacarb and Methoxyfenozide against Laboratory Populations of H. armigera

The LC_50_ values of indoxacarb and methoxyfenozide against laboratory populations of *H. armigera* are presented in Table 1. The populations had different susceptibilities to indoxacarb and methoxyfenozide. Interestingly, indoxacarb toxicity was enhanced 1.83-fold in the MET-SEL *H. armigera*, which showed the highest susceptibility to indoxacarb with an LC_50_ value of 4.43 mg L^−1^. When using MET-SEL *H. armigera* as the reference population, the IND-SEL population showed a low level of resistance to indoxacarb, with a resistance ratio of 8.00. Moreover, susceptibility to methoxyfenozide was increased 2.81-fold in the IND-SEL *H. armigera*, which was more susceptible to methoxyfenozide than the susceptible laboratory population. Compared with the IND-SEL *H. armigera*, the MET-SEL population of *H. armigera* showed a moderate level of resistance to methoxyfenozide, with a resistance ratio of 13.70.

### 3.3. UPLC Method of DCJW Validation

**Figure 2:**
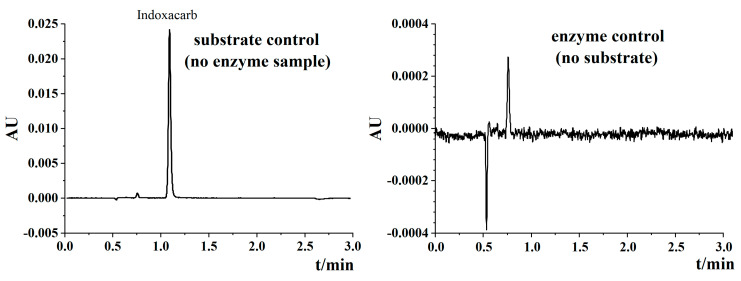
Chromatographs of enzyme control (no substrate) and substrate control (no enzyme sample). Enzyme solution and indoxacarb were needed in the indoxacarb N-Decarbomethoxylation reaction. The chromatographs showed that there was no interfering peak or ghost peak in the enzyme solution control and substrate control.

A recovery experiment with three concentrations (40, 200, and 1000 μg L^−1^) of DCJW was carried out to evaluate the accuracy of the UPLC method. Recovery ratios ranged from 91.56% to 98.39%, and RSDs were in the range of 0.97–3.30% (Table 2). The average recovery ratio of the control was 96.55%, and the LOQ was estimated to be 0.0032 μg L^−1^. No interfering peak or ghost peak was detected in the enzyme solution control (without substrate) (Figure 2). This result indicated that the developed method had good accuracy and precision.

### 3.4. Comparison of N-Decarbomethoxylation Activity among Different H. armigera Populations

**Figure 3:**
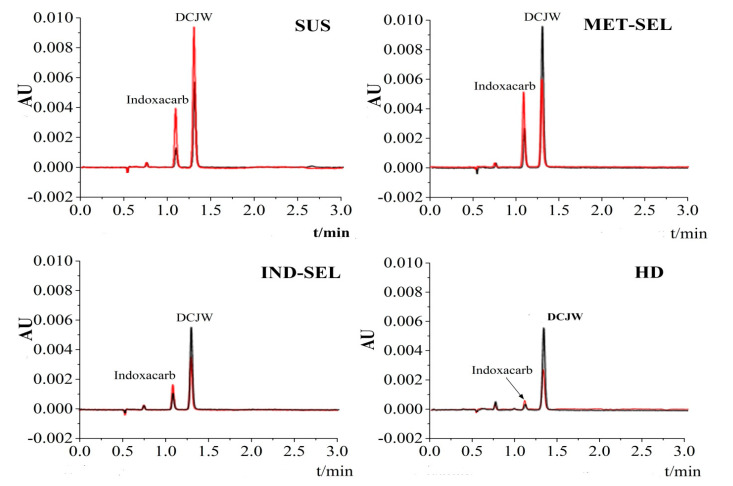
HPLC chromatograms of indoxacarb and DCJW in four populations of *H*. *armigera* from time-course experiments (UV, 314 nm) [9]. Injection volume was 2 μL; indoxacarb and DCJW peaks were integrated. Red line, chromatographic trace for 1 h reactions; black line, chromatogram for reactions that were incubated for 2 h. SUS, susceptible population; MET-SEL, methoxyfenozide-selected population; IND-SEL, indoxacarb-selected population; HD, Handan population.

The peaks of indoxacarb and DCJW in different populations of *H. armigera* are shown in Figure 3. The red line indicates chromatographic trace for 1 h reactions, and the black line represents a chromatogram for reactions that were incubated for 2 h. The retention times of indoxacarb and DCJW were 1.09 and 1.31 min, respectively [9]. Indoxacarb levels decreased over time in all populations, and the amount of DCJW increased in the MET-SEL, IND-SEL, and HD populations throughout the experiment. The highest amounts of indoxacarb and DCJW were retained in the MET-SEL population after 2 h. However, the indoxacarb-resistant (IND-SEL and HD) populations showed significantly lower amounts of indoxacarb and DCJW retention (Table 3). The biotransformation activity of DCJW expressed as pmol (DCJW) mg^−1^ protein min^−1^ is presented in Table 4. The results indicated that DCJW metabolic activation in the SUS and MET-SEL populations was 4.59- and 4.31-fold higher than that in the HD population 1 h after dosing. Moreover, the MET-SEL population had the highest DCJW biotransformation activity 2 h after treatment, but there was no significant difference between the HD, IND-SEL, and SUS populations.

### 3.5. Detoxification-Enzyme Activities

**Figure 4:**
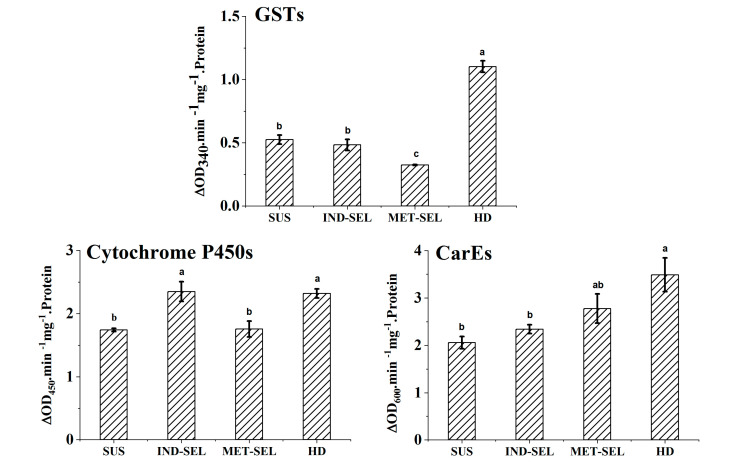
Activities of detoxification enzymes in the midgut of sixth-instar larvae of four populations of *H. armigera*. GST, glutathione S-transferase; Cytochrome P450s, cytochrome P450s mono-oxygenase; CarE, Carboxyl esterase; SUS, susceptible population; MET-SEL, methoxyfenozide-selected population; IND-SEL, indoxacarb-selected population; HD, Handan population. Results are means ± standard error (SE) of three separate replicates. Data marked with different letters were significantly different (*p* < 0.05).

Activities of detoxification enzymes in the midgut of sixth-instar larvae of four populations of *H. armigera* were showed in Figure 4. GST activity in the MET-SEL population was significantly decreased compared with that in other populations (*p* = 0.0001), while GST activity was increased in the HD population. Cytochrome P450 activity was significantly increased in the resistant populations (IND-SEL and HD) relative to activity levels in other populations (*p* = 0.033), while no difference was observed in cytochrome P450 activity between the MET-SEL and susceptible populations. The MET-SEL population showed slightly increased CarE activity, which was significantly increased in the HD population (*p* = 0.014).

## 4. Discussions

Although indoxacarb has high efficacy against lepidopteran pests, *H. armigera* with moderate resistance to indoxacarb was detected in certain areas in China, such as the city of Handan, Hebei province. Our laboratory study suggested that the IND-SEL population was more sensitive to methoxyfenozide than the susceptible population was, and the MET-SEL population showed the highest susceptibility to indoxacarb. The field results also showed that moderately indoxacarb-resistant *H. armigera* were susceptible to methoxyfenozide, and moderately methoxyfenozide-resistant populations were susceptible to indoxacarb. Therefore, methoxyfenozide is a promising insecticide to delay indoxacarb resistance in the field. Similarly, previous studies reported that indoxacarb toxicity was negatively related to methoxyfenozide and abamectin, but positively related to lufenuron and emamectin benzoate in *S. litura* (Lepidoptera: Noctuidae) from Pakistan [22].

The phenomenon in which an insecticide is much more toxic against populations resistant to different insecticides is termed negative cross-resistance. This feature is rare with insecticides, but is more commonly observed with fungicides and herbicides [31]. Cilek et al. demonstrated that diazinon toxicity was enhanced in permethrin-resistant horn flies [32]. In addition, pyrethroid-resistant mosquito populations were reported to be more susceptible to organophosphate insecticides (OPs) than pyrethroid-susceptible strains are [33]. For example, Georghiou et al. reported that temephos resistance in *Culex quinquefasciatus* could be abolished by permethrin resistance and vice versa [34]. Similarly, *C. quinquefasciatus* selection with permethrin abolished pre-existing resistance to malathion. When pyrethroid resistance was increased, the *C. quinquefasciatus* strain was more susceptible to malathion than the susceptible strain was [33]. It was reported that cross-resistance can occur either through changes at the target site (e.g., *N*-methylcarbamates/*N*-propylcarbamates or pyrethroids/*N*-alkylamides) [35,36] that decrease the fitness of resistant strains [37], or through increased metabolic processes, e.g., a pyrethroid-driven increase in the activity of mixed-function oxidases that activate diazinon [32], chlorfenapyr [38], chlorpyrifos, and propoxur [39].

Insecticides used for agricultural pest control undergo physical and enzymatic degradation [10]. The enzyme-associated degradation of insecticides is referred to as “metabolism” or, more appropriately, biotransformation. Detoxification reactions usually lead to the formation of less toxic metabolites with increased polarity [10]. However, the bioactivation of certain insecticides, such as indoxacarb, to apolar and more toxic compounds, has also been reported [6,10,40]. In insects, cytochrome P450s, esterases, and GSTs are the most important detoxification or biotransformation enzymes [41]. Wing et al. demonstrated that the bioactivation of indoxacarb to the toxic DCJW was catalyzed by the enzyme of esterase/amidase because the enzyme could be inhibited by esterase inhibitors DFP (diisopropyl fluorophosphate), paraoxon, and DEF (*S,S,S*-tributyl phosphorothioate), but not by the cytochrome P450s, dependent mono-oxygenase inhibitors piperonyl butoxide (PBO) and 1-phenyl imidazole, or glutathione S-transferase inhibitor N-ethyl maleimide (NEM) [6]. In addition, both indoxacarb and DCJW were further metabolized to relatively more polar hydroxy andoxadiazine ring-opened and hydroxylated ring-opened metabolites in the German cockroach (*Blattella germanica* L.) [10]. The hydroxylated and oxadiazine ring-opened metabolite formation was NADPH/cytochrome P450-dependent. Cytochrome P450 activity was significantly increased in the IND-SEL and HD populations, so a higher disappearance of indoxacarb and DCJW was observed in these two populations. The MET-SEL population showed a slightly increased CarE activity, significantly decreased GST activity, and the highest accumulation of indoxacarb and DCJW. Therefore, it can be concluded that increased CarE activity promoted DCJW formation. The higher accumulation of indoxacarb and DCJW in the MET-SEL population enhanced indoxacarb toxicity.

Resistance to indoxacarb was reported to be related to P450s, esterases, and GSTs in many insect pests [18,21,42,43]. Ahmad et al. reported that increased oxidase detoxification is a major resistance mechanism to indoxacarb because PBO could reduce indoxacarb resistance from 705- to 20-fold in a resistant field population of *Choristoneura rosaceana* [43]. Meanwhile, indoxacarb toxicity was increased by PBO and DEM (diethyl maleate), reflecting that enhanced P450 and GST activities played important roles in the indoxacarb resistance of a highly resistant *P. xylostella* population (resistance ratio = 1069.3) [21]. Moreover, it was reported that improved metabolisms by P450s and esterases were the main detoxification mechanism responsible for resistance to indoxacarb in a highly resistant *P. xylostella* population [14]. On the one hand, indoxacarb is activated through hydrolysis by esterase; on the other hand, resistance to indoxacarb is esterase-associated. Therefore, different esterases are involved in the activation and detoxification processes. Significantly increased P450s, esterases and GST activities in the resistant HD field population strongly suggested that indoxacarb resistance in the field population was associated with increased metabolism by P450s, GSTs, and esterases. The improved P450 metabolism was the main resistance mechanism to indoxacarb in the IND-SEL population.

In summary, our results suggest that there is a negative correlation between methoxyfenozide and indoxacarb resistance. The improved toxicity of indoxacarb, as observed in the MET-SEL *H. armigera*, resulted from the higher accumulation of indoxacarb and DCJW. The significantly increased activity of cytochrome P450s and the higher levels of disappearance of indoxacarb and DCJW in the indoxacarb-resistant populations (IND-SEL and HD) resulted in decreased toxicity of indoxacarb against *H. armigera*. In addition, the strengthened activities of cytochrome P450s, CarEs, and GSTs may have contributed to the moderate resistance to indoxacarb in field *H. armigera*. This study provides useful information on indoxacarb resistance mechanisms and suggests that methoxyfenozide can be rotationally used to manage indoxacarb resistance.

## Figures and Tables

**Table 1:** Resistance of susceptible (SUS), indoxacarb-selected (IND-SEL) and methoxyfenozide-selected (MET-SEL) *H. armigera* populations to indoxacarb and methoxyfenozide.

Population	Insecticide	Slope (±SE)	LC_50_ (95% CL) (μg mL^−1^)	N *^a^*	Indoxacarb RR *^b^*	Methoxyfenozide RR *^c^*
SUS	Indoxacarb	1.52 ± 0.092	8.12 (6.70–9.80)	210	1.83	
	Methoxyfenozide	1.11 ± 0.105	83.73 (69.19–101.32)	210		2.81
IND-SEL	Indoxacarb	1.52 ± 0.020	35.42 (34.23–36.65)	210	8.00	
	Methoxyfenozide	1.74 ± 0.097	29.79 (25.99–34.15)	210		1.00
MET-SEL	Indoxacarb	1.05 ± 0.054	4.43 (3.63–5.41)	210	1.00	
	Methoxyfenozide	1.08 ± 0.075	408.04 (356.03–467.66)	210		13.70

CL: confidence limit; *^a^* number of larvae used in bioassay; *^b^* indoxacarb resistance ratio (RR) = LC_50_ (SUS or IND-SEL)/LC_50_ (MET-SEL); ^*c*^ methoxyfenozide RR = LC_50_ (SUS or MET-SEL)/LC_50_ (IND-SEL).

**Table 2:** Accuracy and precision of proposed method to detect DCJW in *H. armigera* at three spiked levels (*n* = 3). RSD, relative standard deviation.

Applied (μg L^−1^)	Found (μg L^−1^)	Recovery Ratio (%) ± SE	RSD (%)
40	39.1	98.39 ± 0.89 a	1.55
200	181.6	91.82 ± 1.90 b	3.30
1000	915.6	91.56 ± 0.56 b	0.97

Data presented as mean ± standard error (SE). Data marked with different letters were significantly different (*p* < 0.05).

**Table 3:** The concentrations of DCJW in four populations of *H. armigera.*

*H. armigera* Population	Concentration of DCJW (μg L^−1^)		
	N ^a^	1 h	2 h
SUS	30	353.45 ± 17.07 a	224.01± 7.32 b
MET-SEL	30	442.36 ± 57.94 a	474.41± 26.54 a
IND-SEL	30	207.28 ± 9.35 b	263.33 ± 21.37 b
HD	30	97.30 ± 9.04 b	150.39 ± 23.62 b

^a^ number of larvae used in test. Data marked with different letters were significantly different (*p* < 0.05).

**Table 4:** Comparison of indoxacarb N-decarbomethoxylation activity between four *H. armigera* populations.

*H. armigera* Population	N-Decarbomethoxylation Activity (pmol (DCJW) mg^−1^ protein min^−1^) (Mean ± SE)				
	N ^a^	1 h	Activity Ratio	2 h	Activity Ratio
SUS	30	7.39 ± 0.21 a	4.59	2.13 ± 0.31 b	0.86
MET-SEL	30	6.94 ± 0.08 a	4.31	4.19 ± 0.23 a	1.68
IND-SEL	30	3.14 ± 0.42 b	1.95	1.98 ± 0.11 b	0.79
HD	30	1.61 ± 0.13 b	1	2.49 ± 0.40 b	1

^a^ number of larvae used in test; Activity ratio = N-decarbomethoxylation activity of other populations/N-decarbomethoxylation activity of HD population. Results are mean ± standard error (SE) of three separate replicates. Data marked with different letters were significantly different (*p* < 0.05). SUS, susceptible population; MET-SEL, methoxyfenozide-selected population; IND-SEL, indoxacarb-selected population; HD, Handan population.
